# In planta engineering of polysialylated glycoproteins using salmonid polysialyltransferases

**DOI:** 10.1038/s41598-026-52259-3

**Published:** 2026-05-11

**Authors:** Lin Sun, Anna Seidel, Hauke Thiesler, Jennifer Schoberer, Stanislav Melnik, Alexandra Castilho, Herbert Hildebrandt, Anne Harduin-Lepers, Sebastian P. Galuska, Richard Strasser, Rita Gerardy-Schahn, Herta Steinkellner, Somanath Kallolimath

**Affiliations:** 1 Institute of Plant Biotechnology and Cell Biology, Department of Biotechnology and Food Sciences, BOKU University, Muthgasse 18, 1190 Vienna, Austria; 2 Research Institute for Farm Animal Biology (FBN), Wilhelm-Stahl-Allee 2, 18196 Dummerstorf, Germany; 3 Hannover Medical School, Institute of Clinical Biochemistry, Carl-Neuberg-Str. 1, 30625 Hannover, Germany; 4 Center for Systems Neuroscience Hannover (ZSN), 30625 Hannover, Germany; 5 Univ. Lille, CNRS, UMR 8576 - UGSF - Unité de Glycobiologie Structurale et Fonctionnelle, 59000 Lille, France

**Keywords:** Salmonid polysialyltransferases, Polysialic acid, *Nicotiana benthamiana*, N-glycan engineering, NCAM, VEGFR, Biochemistry, Biological techniques, Biotechnology, Plant sciences

## Abstract

Polysialic acid (polySia) is a glycan polymer composed of a linear chain of sialic acid residues detected on a small set of proteins in mammalian cells. This negatively charged glycan modulates various cellular functions and has potential biomedical applications. However, the targeted synthesis of polySia remains challenging due to biosynthetic and structural complexity. In this study, we investigated three polysialyltransferases from the salmonid *Coregonus maraena* (Cma-ST8Sia) for their application in glycoengineering. For this, the corresponding genes *Cmast8Sia2-R1*,* Cmast8Sia2-R2*, and *Cmast8Sia4* were transiently expressed in *Nicotiana benthamiana*, and confocal laser scanning microscopy of GFP-fused Cma-ST8Sia exhibited trans-Golgi localization. Co-expression of Cma-ST8Sia with genes from the mammalian sialic acid biosynthesis pathway resulted in autopolysialylation of CmaST8SiaII-R1 and CmaST8SiaIV, but not of CmaST8SiaII-R2. Furthermore, co-expression with glycoproteins demonstrated polysialylation of recombinant proteins with distinct efficiencies and chain lengths. Inactive EndoN-coupled resin was used to enrich polysialylated proteins, and by the application of HPLC/DMB labeling, a degree of polymerization up to 45 was determined. Finally, a microglia inhibition assay demonstrated the biological activity of the engineered polySia. Collectively, these findings advance the capacity to engineer high-quality polySia in an eco-friendly, sustainable system, facilitating in-depth studies and the rational design of complex glycans.

**Supplementary Information:**

The online version contains supplementary material available at 10.1038/s41598-026-52259-3.

## Introduction

Polysialic acid (polySia) is a glycan polymer composed of large linear chains of α2,8-linked sialic acid residues (N-acetyl neuraminic acid; Neu5Ac), found on N-linked or O-linked glycans of a small set of glycoproteins in mammalian cells. This highly negatively charged and restricted modification controls crucial biological processes such as neuronal development, plasticity, and regeneration^1^. PolySia also plays a role in tumor biology^2,3^ and modulates immunological processes like neutralizing extracellular histones and neutrophil traps^4^, dendritic cell trafficking via electrophilic interactions with central chemokine receptors and ligands^5,6^. It also reduces inflammation by inhibiting nitric oxide and pro-inflammatory cytokine production via immune receptors of the Siglec family in macrophages and microglia, and possibly by interfering with complement activation^7^. Its interactions with vascular endothelial growth factor A-188 affect glomerular microvasculature and nephrogenesis^8^. PolySia was shown to stabilize protein drugs to improve pharmacokinetics^9,10^. Owing to its non-immunogenicity and hydrophilicity, it is suitable as a scaffold material for tissue engineering applications^11,12^. In addition, polySia-based nanocarriers have been developed for targeting the central nervous system, as they are capable of crossing the blood-brain barrier^13^.

In mammals and lower vertebrates, synthesis of sialic acid polymers is catalyzed by polysialyltransferases ST8Sia (CAZy family GT29). In humans, ST8SiaII and ST8SiaIV synthesize sialic acid polymers exclusively with α2,8 linkages^14^. In contrast, cold-blooded vertebrates and pathogenic bacteria exhibit additional linkage types, including α2,9 and alternative α2,8/α2,9, and incorporate various sialic acids such as N-glycolyl neuraminic acid (Neu5Gc) and deaminated sialic acid (KDN), alongside Neu5Ac^15–17^. The degree of polymerization (DP) varies tremendously from a few to several hundred sialic acid residues^18^. Interestingly, the DP in non-vertebrate deuterostomes is more restricted. For example, glycomics studies in Echinodermata have revealed short-chain polySia (DP4–40)^19–21^. Evolutionary analysis reveals widespread ST8Sia activity in both vertebrates and invertebrates, with high diversity in substrate specificity and chain length. In particular, teleost (bony fish) genomes exhibit a heterogeneous pattern; some ST8Sia genes are duplicated, while others are absent, suggesting a dynamic molecular evolution of their functional diversification^14,22^. Notably, it has been suggested that salmonid ST8Sias synthesize shorter polySia chains than mammalian enzymes, supported by the detection of short polySia DP < 26 in salmonid fish egg polysialo-glycoprotein and by in vitro polysialylation^23,24^.

Interestingly, the DP of polySia determines its biological activities. For example, the therapeutic effects of polySia in a mouse model of macular degeneration were observed with an average DP20^25^, and a minimal DP24 was required for an effective inhibition of microglial inflammatory activity^26^. The binding of brain-derived neurotrophic factor and fibroblast growth factor 2 requires a minimum DP of 12 and 17, respectively^27^. DP12 fragments have been applied to restore synaptic and cognitive functions of polySia-deficient mice and in dementia mouse models^28^.

Due to its multiple functions, the application of polySia in the pharmaceutical field is gaining increasing importance. However, the limited availability of polySia in high quality and possibly with a defined chain length remains a significant barrier to comprehensive functional studies. Moreover, most analytical systems are not optimized for detecting such complex and highly unstable carbohydrates. Current polySia production by pathway engineering and in vitro glycoengineering has led to remarkable success^29,30^ but has not yet led to the generation of sufficient quality material for in vivo applications.

As shown in a previous study, human ST8SiaII and ST8SiaIV can synthesize polySia in heterologous expression systems, covering a wide range of DPs^29^. In contrast, salmonid ST8Sias have been proposed to produce polySia with restricted chain lengths, making these enzymes interesting for glycan engineering^23^.

In this study, we focused on characterizing polysialyltransferases from the salmonid fish *Coregonus maraena* (Cma-ST8Sia) for their ability to synthesize polySia in a heterologous plant-based system. The three known Cma-ST8Sia genes were transiently co-expressed in plants together with genes for the mammalian sialic pathway (mSAP). The ability of Cma-ST8Sias to perform auto- and trans-polysialylation was evaluated. Additionally, we optimized the isolation of polysialylated proteins and showed relevant functional activity.

## Results and discussion

### *In planta* expression of Cma-ST8Sia

**Fig. 1:**
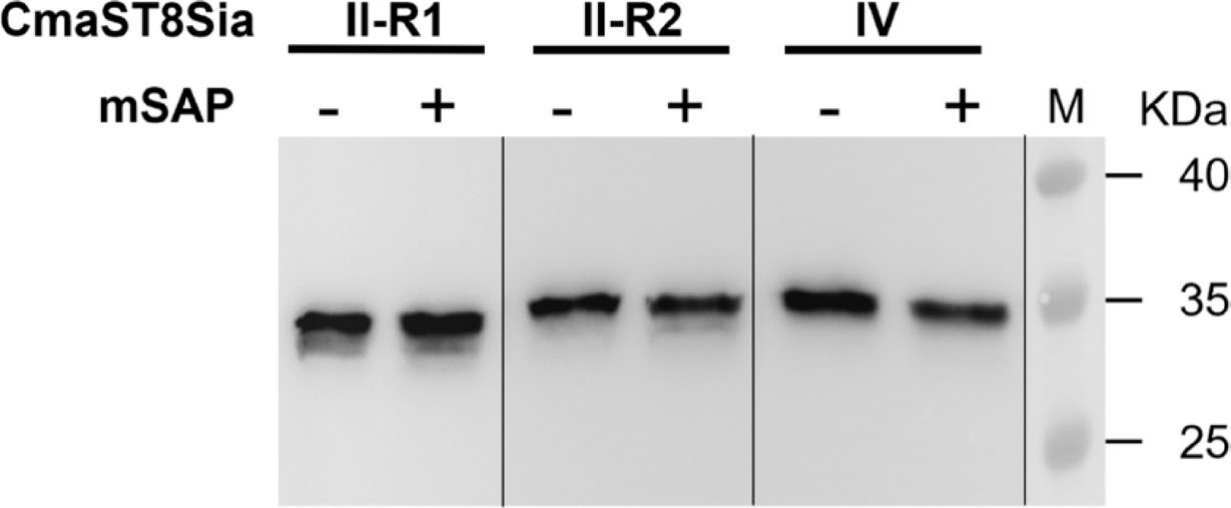
Detection of Cma-ST8Sia expression in *N. benthamiana* by Western blot analysis. Total protein extracts from ΔXTFT leaves infiltrated with Cma-ST8Sia (II-R1, II-R2, or IV), without (-) or with (+) mammalian sialylation pathway (mSAP), were monitored for expression of Cma-ST8Sia using anti-strep antibodies. Approximately 50 µg of protein was loaded per lane. The protein ladder (M) is indicated in KDa.

In this study, we used ΔXTFT, glycan-engineered *N. benthamiana* plants that lack β1,2-xylosylation and core α1,3-fucosylation^31^, for recombinant expression of glycoproteins. This line is a widely used expression and engineering platform^32^ and has served as a host for engineering polySia using human ST8SiaII and IV^29^. Respective DNA vectors carrying genes from the three salmonid Cma-ST8Sia (CmaST8SiaII-R1, CmaST8SiaII-R2, and CmaST8SiaIV) were delivered into plant leaves with and without mSAP by agroinfiltration. Enzyme expression was monitored three days post-infiltration (dpi) in total protein extracts (TP) by Western blotting (Fig. 1). Specific signals at ~ 35 KDa were detected and assigned as recombinant Cma-ST8Sia. Expression of all three enzyme variants was evenly detected. No differences in expression levels were observed by co-expression with mSAP, which contrasts with human ST8Sias, where co-expression of mSAP has been linked to enhanced expression *in planta*^29^. No signals were detected in the intercellular fluid (apoplast), representing the plant secretome, indicating that the recombinant enzymes were intracellularly located (Figure S4A).

### Subcellular localization of Cma-ST8Sia

**Fig. 2:**
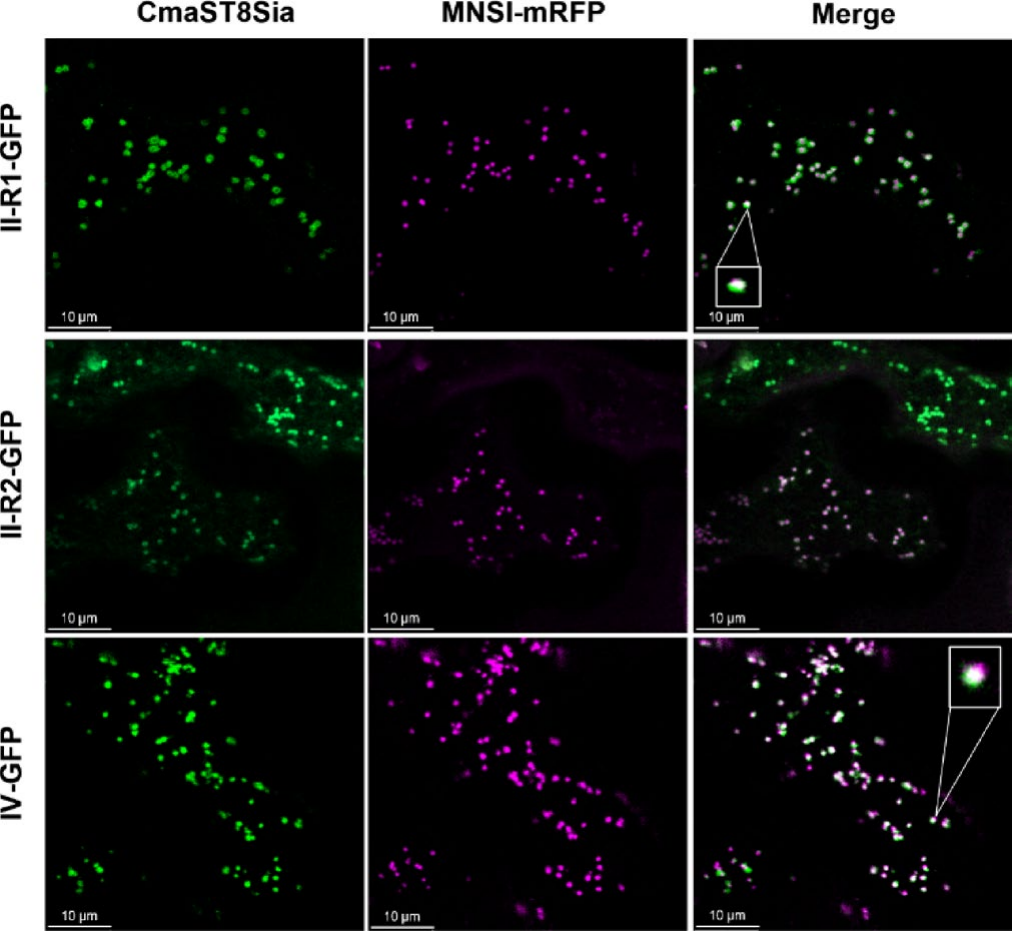
Subcellular localization of the Cma-ST8Sia-GFP. Representative confocal images of leaf epidermal cells co-expressing Cma-ST8Sia-GFP fusions (pCmaST8-II-R1-, II-R2-, or IV-GFP, Figure S2A) and the Golgi marker MNSI-mRFP (mRFP: monomeric red fluorescent protein). The punctate staining pattern of Cma-ST8Sia-GFP (green), which overlaps with that of MNSI-mRFP (magenta), indicates Golgi localization (merge) and appears in white. Square shows the magnification of a single Golgi stack. Scale bars = 10 μm.

Confocal laser scanning microscopy (CLSM) was used to determine the precise subcellular localization of recombinant Cma-ST8Sia. For this purpose, Cma-ST8Sia-GFP fusions were transiently co-expressed in ΔXTFT leaves with Golgi-mannosidase I fused to mRFP (MNSI-mRFP), a well-characterized Golgi marker, located in cis-medial cisternae^33^. Confocal images revealed a punctate staining pattern characteristic of Golgi bodies in plant cells^34^. Importantly, GFP signals colocalized with the MNSI-mRFP, indicating Golgi localization of all three Cma-ST8Sia (Fig. 2 and Figure S11). Close-up inspection of fluorescent signals exhibited partial colocalization of Cma-ST8Sia-GFP and MNSI-mRFP, indicating distinct sub-Golgi localization. As MNSI-mRFP is a well-established *cis-medial* marker, a trans-Golgi deposition is anticipated for Cma-ST8Sia-GFP. This finding is consistent with previous studies that show trans-Golgi retention of human STs in plants^29,35^. Glycan maturation steps are a highly orchestrated process along the sub-Golgi compartments; even a slight shift of enzymes within Golgi cisternae can significantly alter the final glycosylation profile^36,37^. Although some Golgi targeting and retention motifs are conserved across eukaryotic species, the precise molecular mechanisms governing localization/retention are not fully understood^38,39^.

### *In planta* autopolysialylation of Cma-ST8Sia

**Fig. 3:**
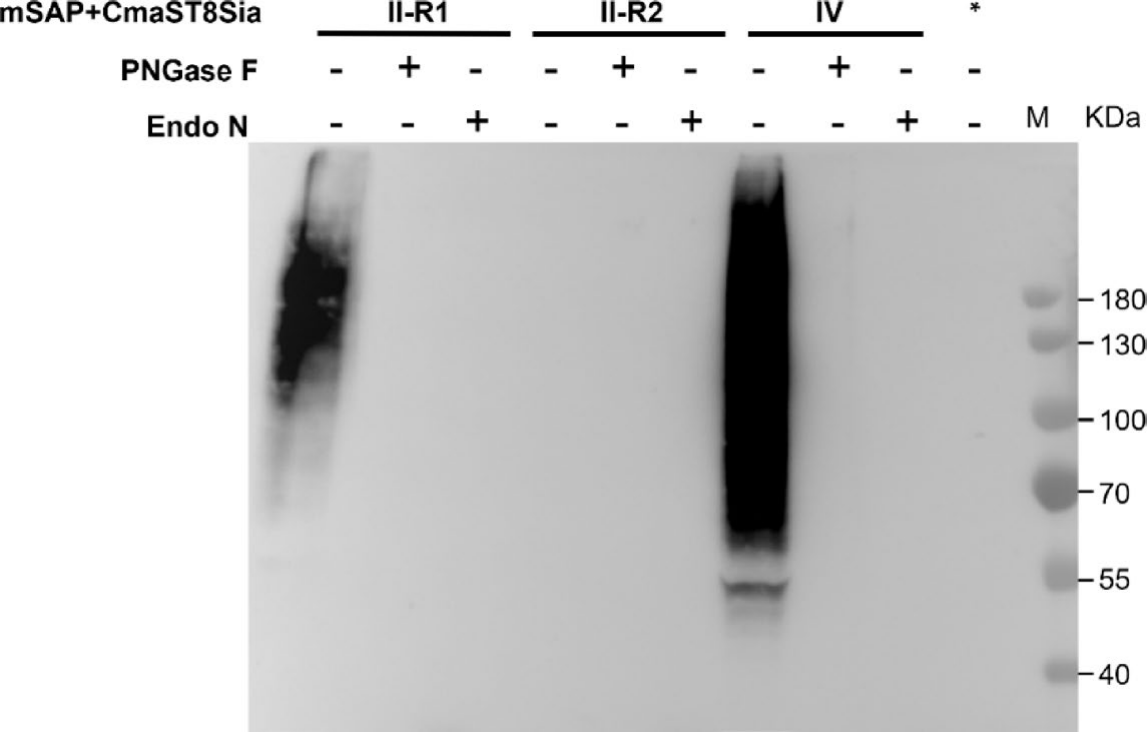
Detection of Cma-ST8Sia autopolysialylation by Western blot analysis. ΔXTFT leaves were co-infiltrated with Cma-ST8Sia and mSAP. The total protein extracts (TP) were monitored using anti-polySia antibody mAb735: ~50 µg TPs were loaded per lane. TP was treated with (+) or without (-) PNGase F and endoneuraminidase N (EndoN), respectively. (*) mSAP without Cma-ST8Sia.

To investigate *in planta* autocatalytic activity, Cma-ST8Sia were co-expressed with mSAP. TP was analyzed by Western blot using the anti-polySia monoclonal antibody mAb735^40^. PolySia-specific smear signals were detected for CmaST8SiaII-R1 and -IV, with higher intensity of the latter (Fig. 3). The broad staining range of CmaST8SiaIV from 60 KDa onwards indicates a larger chain length distribution than that of -R1, whose signal range is between 80 and 200 KDa. No signal was detected for CmaST8SiaII-R2. Further characterization of polySia was performed by incubation of TP with EndoN, an enzyme that specifically cleaves α2,8-linked polySia, and PNGaseF, an N-glycan-specific endoglycosidase. Both treatments resulted in the abolishment of the staining pattern, confirming the presence of α2,8-linked polySia on N-glycans (Fig. 3). Collectively, the results indicate autopolysialylation of CmaST8SiaII-R1 and IV. No polySia-specific signals were observed when IF was monitored, indicating exclusive intracellular deposition of the enzymes (Figure S4B).

Autopolysialylation of human ST8Sias has been reported previously^41,42^. Interestingly, human ST8Sias and CmaST8SiaIV show a similarly broad autopolysialylation staining, but differ from the relatively narrow staining of CmaST8SiaII-R1^29^. The lack of autocatalytic activity for CmaST8SiaII-R2 came as a surprise, considering its high sequence similarity with CmaST8SiaII-R1 including conservation of all N-glycosylation sites. In fact, the polysialyltransferase domain, involved in polysialylation activity of enzymes, is identical (Figure S1^43^, . One reason could be that CmaST8SiaIIR2 has a different donor sugar preference for its activity, such as Neu5Gc or KDN. No other sialic acid variants than Neu5Ac are synthesized in our engineered plants. Currently, the impact of autopolysialylation is not entirely clear, but it appears that at least some ST8Sias require it for enzymatic activity and efficient elongation of polySia chains on selective glycoproteins^44^. It also may serve as a stabilization factor^44,45^ and enhance expression levels in plants^29^.

### Recombinant expression and glycan analysis of reporter proteins

To test whether Cma-ST8Sias can polysialylate recombinant proteins, two reporter proteins were selected. Ig5FN1 is a well-characterized domain of human NCAM known to carry polySia^46,47^. In previous studies, Ig5FN1 served as a model for *in planta* engineering of polySia using human ST8SiaII and IV^29^. VEGFR fused to monomeric IgG1 Fc (VEGFRmFc) served as an additional reporter. It is a highly sialylated glycoprotein that is used in its dimeric form (i.e., Aflibercept) for therapeutic purposes^48^. Highly sialylated Aflibercept was recombinantly produced in plants and mammalian cells^49,50^. Although not specifically tested for polysialylation, there is no evidence suggesting this modification. Respective DNA constructs that direct Aflibercept, VEGFRmFc, and Ig5FN1 to the secretory pathway and finally to the apoplast were delivered into ΔXTFT leaves with and without mSAP. SDS-PAGE analysis confirmed the presence of recombinant proteins in the apoplast (Figure S5A and B). Interestingly, VEGFRmFc exhibited less degradation compared to aflibercept and is thus used for further studies. Stabilization of proteins by using monomeric IgG-Fc fusions has been reported previously^51^.

The Ig5FN1 and VEGFRmFc carry three and five N-glycosites (GS), respectively (Figure S6). A precise glycosylation profile for individual GS was determined by mass spectrometry analysis (LC-ESI-MS). Ig5FN1 sialylation content of GS1, 2, and 3 was about 52, 63, and 69%, respectively (for details see Table S2). VEGFRmFc exhibits an overall sialylation content of 66, 47, 48, 97, and 34% for GS1-5, respectively (for details, see Table S3). Collectively, the data exhibits that the in-planta engineering approach resulted in the generation of reporter proteins with a high degree of sialylation, providing suitable acceptor substrates for polysialylation.

### Polysialylation activity of Cma-ST8Sia on reporter proteins

**Fig. 4:**
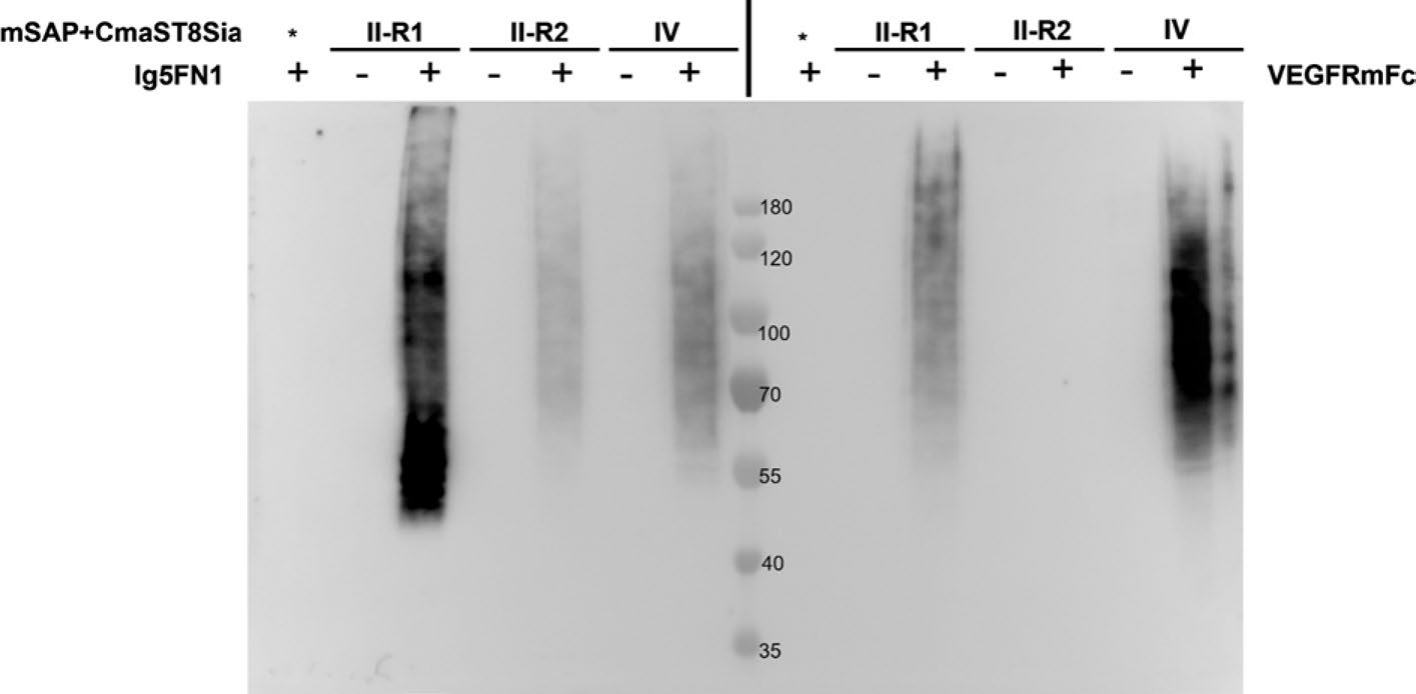
Detection of polysialylated Ig5FN1 and VEGFRmFc by Western blot analysis using the mAb735 antibody. Intercellular fluid (IF) isolated from leaf infiltrated with mSAP, Cma-ST8Sia with (+) and without (-) Ig5FN1 (left) and VEGFRmFc (right); 10 µL of IF was loaded per lane. (*) mSAP without Cma-ST8Sia. See also Figure S7.

In a next set of experiments, Ig5FN1 and VEGFRmFc were co-expressed with mSAP and Cma-ST8Sia in ΔXTFT. IF was collected at 4 dpi, and expression of reporter proteins was confirmed by SDS-PAGE (Figure S5C). Notably, in contrast to proteins derived from TP, IF does not contain Cma-ST8Sia, as the enzymes are retained in the Golgi apparatus. Thus, mAb735 signals are assigned to the reporter proteins, targeted to the apoplast. Western blot analysis of IF exhibited signals upon co-expression of CmaST8SiaII-R1 and IV for both recombinant proteins, albeit with different intensities and molecular masses (Fig. 4). Comparing signal intensities, it appears that CmaST8SiaII-R1 polysialylates Ig5FN1 more efficiently, while CmaST8SiaIV prefers VEGFRmFc (Fig. 4). Regarding the molecular mass, it appears that CmaST8SiaII-R1 synthesizes a shorter chain length on Ig5FN1 compared to all other enzyme/ reporter protein combinations. By contrast, CmaST8SiaII-R2 inefficiently polysialylates Ig5FN1 but not VEGFRmFc. No signal was detected in the IF without reporter protein co-expression, indicating that the signal is specific for polysialylation of the reporter and not associated with autopolysialylation. To reconfirm the absence of autopolysialylated CmaST8Sias in IF, we conducted an anti-strep Western blot following endo N enrichment of IF samples containing VEGFRmFc, mSAP, and CmaST8Sias. The results (Figure S9) clearly indicate no leakage of autopolysialylated CmaST8Sias into IF. The sharp bands at ~ 75 kDa on the anti-strep blot of the Endo N-treated sample represent Endo N, which carries a strep-tag used for affinity purification.

In summary, the CmaST8SiaII-R1 and IV are capable of synthesizing polysialylated N-glycans on reporter proteins, however, with distinct efficiencies. Notably, the ability to synthesize polysialylated proteins beyond known natural substrates is not known for mammalian enzymes. However, it has been observed for human ST8Sias used in plant engineering (https://patents.google.com/patent/WO2018069279A1/en). Whether expansion of substrate recognition is associated with plant engineering or a broader phenomenon is currently not known and needs further investigation.

The fact that the two autopolysialylated CmaST8Sias are more active than CmaST8SiaII-R2, for which no autopolysialylation could be detected, indicates that this modification affects enzyme activity. The role of autopolysialylation for certain functional properties has been observed using human ST8Sias, but is not yet fully understood^44,45^. A critical role for ST8Sia autopolysialylation in substrate selection and chain elongation has been suggested^44^. In terms of DP, CmaST8SiaIV behaves most similarly to human ST8SiaIV, which synthesizes long polySia chains^52^. The most striking difference was observed for polysialylation of Ig5FN1 by CmaST8SiaII-R1, which shows strong signals between 50 and 60 KDa. The reasons with this relatively short DP are currently unknown.

The polysialyltransferases are highly specific, and only a limited number of glycoproteins are modified by these enzymes. The VEGFRmFc has not been tested as a substrate for polysialylation activity before. By considering the differences between mammalian and plant expression systems, such as the absence of the sialic acid salvage pathway and sialidases in plants, we cannot rule out the effects of using different expression systems on polysialylation. In this context, we highlight that these expression system-specific differences could positively influence the polysialylation reaction, potentially enhancing the modification of highly sialylated non-natural glycoproteins by polysialyltransferases.

### Enrichment of polysialylated proteins by inactive EndoN

A major obstacle in polySia research is obtaining sufficient high-quality material. Here, we used inactive EndoN generated by introducing a mutation in its active site; as a consequence, it can bind polySia but cannot degrade it^53^. Inactive EndoN-coupled CNBr resin was incubated with IF containing polysialylated Ig5FN1 and VEGFRmFc. All fractions were subjected to immunoblotting using anti-polySia mAb 735, and elution fractions, which correspond to 50-fold enrichment, exhibited a significantly enhanced signal (Figure S8 and S9). Next, elution fractions were treated with active EndoN to confirm polySia on the protein-bound N-glycans. Immunoblotting, using protein-specific mAbs, shows a distinct staining pattern, with a shift towards the expected protein size. The results indicate the presence of polySia linked to N-glycans on reporter proteins (Figure S8 and S9). In summary, the results show efficient enrichment of polySia proteins by inactive EndoN.

Due to a high negative charge, polySia can also be enriched using an ion exchange chromatography. This involves the risk of binding to all negatively charged molecules, including non-polysialylated proteins. Alternatively, the mAb735 antibody, which requires DP8 or more for its binding, was also used to enrich polySia^54^. EndoN exhibits high stability across a broad pH range (4 to 12) and requires a minimum degree of polymerization (DP5) for binding to polySia^55,56^. These properties make it highly suitable for affinity enrichment applications^23,57^.

### Chain length analysis of polysialic acid

**Fig. 5:**
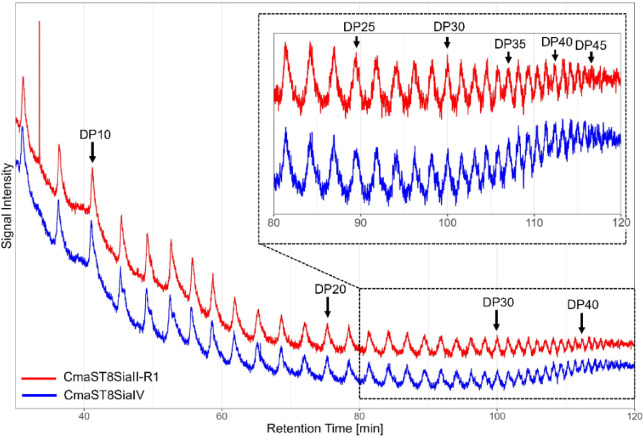
PolySia DP-analysis by DMB-anion exchange HPLC. VEGFRmFc derived from leaves co-infiltrated with mSAP, CmaST8SiaII-R1 (red line) or CmaST8SiaIV (blue line); IF was precipitated using inactive Endo N coupled to magnetic beads and subsequently analyzed by DMB-anion exchange HPLC. For background compensation, the chromatogram of a run with only DMB reagent without any polySia was subtracted from the sample runs. The DP of selected peaks is given.

To investigate polySia DP, an anion exchange HPLC approach, using DMB as fluorescence label, was performed. To this end, IF containing polysialylated VEGFRmFc was enriched by inactive EndoN-coupled to magnetic beads, subsequently released and fluorescently labeled for HPLC analysis. Colominic acid, a bacterial-derived polySia, was used as a standard to assign DP (Figure S10A). The obtained chromatograms revealed chains consisting of at least 45 sialic acid residues for both CmaST8SiaII-R1 and CmaST8SiaIV (Fig. 5). A higher DP than 45 is anticipated since a peak elutes at the start of the wash cycle with high salt concentrations at 130 min (Figure S10B and C). As discussed in a previous study, internal strand breaks also occur during the one-pot reaction under acidic conditions, in which polySia chains are released and fluorescently labeled^58^. The presence of DP larger than 45 is also supported by Western blot analysis, which shows intense polySia-specific signals at a position above the expected molecular mass of the two reporter proteins (Fig. 4 and S7). Importantly, the unequivocal presence of up to DP 45 enables further binding experiments to all described polySia interaction partners^3,7^.

Our results regarding size distribution and estimated DP are similar to plant-based engineering using human ST8Sias^29^. A DP of 45 and possibly above came as a surprise since short-chain polySia, i.e., DP5–25, has been detected in salmonid fish egg glycoproteins and in engineering approaches using mammalian cells^16,23,24^. Our results indicate that short DP is not a consequence of enzymatic activity, but rather may be due to the incorporation of sialic acid analogues, like KDN. KDN is known to act as a stop signal for chain elongation and has been found as the terminal sialic acid of polySia in lower vertebrates, including salmonids^59^. The involvement of highly active sialidases or competition with other glycosylation pathways cannot be ruled out.

### Functional activity of plant-produced polySia

**Fig. 6:**
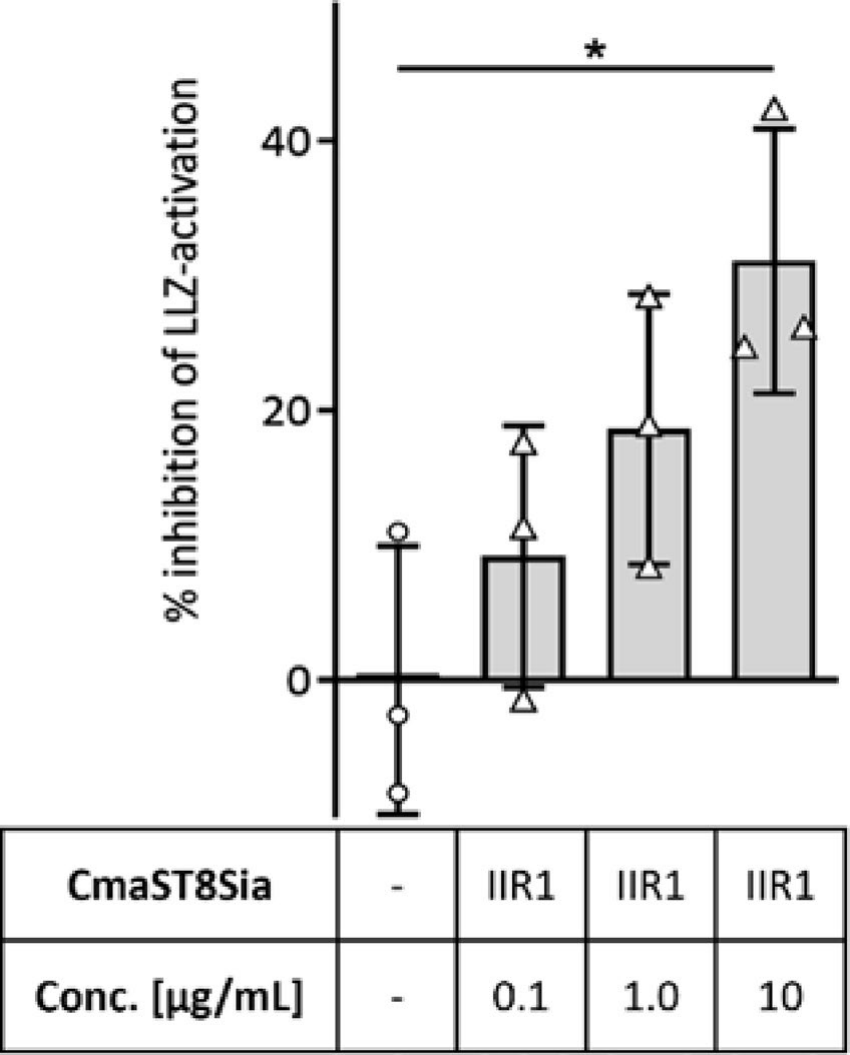
Determination of polySia activity using a microglia inhibition assay. Nitric oxide production of BV-2 microglia cultured in the presence of 10 ng/mL of LPS, 50 ng/mL of LPS-binding protein, and 10 µg/mL of zymosan (LLZ); in serum-free medium and treated without (–) or with the indicated amounts of 0.1–10 µg/mL of polysialylated VEGFRmFc produced by co-expression of CmaST8SiaII-R1. One-way ANOVA indicated significant differences (*p* = 0.025), and results from Tukey’s post hoc test are shown (*, *p* < 0.05).

Finally, IF-derived polysialylated VEGFRmFc was investigated for its capability to inhibit microglia activation, an activity specifically mediated by polySia structures^60^. The results show that plant-derived polySia is potent in inhibiting LLZ-induced nitric oxide production of BV2 microglia in a concentration-dependent manner. An inhibition of ∼30% was achieved in the presence of 10 µg/mL polysialylated VEGFRmFc (Fig. 6). Previous studies show a similar inhibition of LLZ-activated BV2 microglia using 4 µg/mL (i.e., 250 nM) of colominic acid (bacterial-derived polySia) with an average DP of ∼50^26^, and of LPS-induced primary and stem-cell-derived microglia treated with up to 50 µg/mL of colominic acid^61,62^, indicating that the anti-inflammatory effect of plant-produced polySia is as potent as that of colominic acid. The results are consistent with a previous study using plant-derived Ig5FN1-polySia^29^. Thus, by using an *in planta* glycoengineering approach, the plants can efficiently synthesize polySia on pharmaceutically relevant proteins that are not polysialylated under natural conditions, and plant-derived polySia is functionally active.

## Conclusion

In recent years, there has been an increasing demand for the generation of environmentally friendly, biodegradable biomaterials derived from sustainable sources. In this study, we report the production of polysialic acid (polySia), a biomedically interesting biopolymer, through a sustainable, plant-based engineering system. We are expanding the repertoire/toolbox for the development of high-quality polySia using enzymes from sources evolutionarily distant from those of mammals. The results suggest that there are many other enzymes with polySia activity in nature that could be of interest for targeted engineering, especially for controlling DP. Despite our considerable success, some important questions remain unanswered. For example, why do CmaST8SiaII-R1 and -R2 exhibit distinct auto- and trans-polysialylation activities despite high sequence similarity? What are the underlying molecular mechanisms of CmaST8Sia substrate specificity, especially beyond known natural substrates? Further experiments are needed to better understand and subsequently control polySia synthesis. This study provides valuable results in this regard.

## Materials and methods

### Construction of expression vectors

cDNAs encoding three Cma-ST8Sia genes, *st8Sia2-R1*,* st8Sia2-R2*, and *st8Sia4*, originating from the *C. maraena*^23^, were PCR amplified with primer pairs (Table S1) carrying terminal restriction sites *Xba*I*/Bgl*II (*st8Sia2-R1* and *st8Sia2-R2*) or *Xba*I*/BamH*I (*st8Sia4*). Digested, PCR products were cloned into a plant expression vector carrying a C-terminal strepII- and GFP-tag, respectively^29^. The resulting six vectors pCmaST8SiaII-R1-Strep, pCmaST8SiaII-R2-Strep, pCmaST8SiaIV-Strep, pCmaST8SiaII-R1-GFP, pCmaST8SiaII-R2-GFP, and pCmaST8SiaIV-GFP (Figure S1 and S2A) were transformed into *Agrobacterium tumefaciens* (strain GV3101 pMP90).

The synthetic cDNA encoding vascular endothelial growth factor (VEGF) receptor R1 and R2 domains codon optimized for *N. benthamiana* was ordered from BioCat GmbH (https://www.biocat.com/) with *Bsa*I flacking sites and subsequently cloned by Golden Gate assembly into the magnICON^®^ vector carrying N-terminal α-amylase signal peptide for secretion, C-terminal monomeric Fc for detection and purification^51^. The resulting expression vector for the VEGFRmFc was transformed into *A. tumefaciens* (strain GV3101 pMP90). The expression vector for Ig5FN1 (accession: KU052570.1) was described previously^29^, and the amino acid sequence of VEGFRmFc was shown in (Figure S2B and Figure S3).

### *In planta* transient protein expression

Agrobacterium carrying genes of Cma-ST8Sia and genes involved in mammalian sialic acid pathway (mSAP: GNE: mouse UDP-GlcNAc 2-epimerase/N-acetylmannosamine kinase, NANS: human N-acetyl neuraminic acid synthase, CMAS: human CMP-sialic acid synthase, CST: mouse CMP-sialic acid transporter, ^ST^GalT: human β1,4-galactosyltransferase, ST: rat α2,6-sialyltransferase) were transiently co-expressed in *Nicotiana benthamiana* ∆XTFT plants^31^.

For agroinfiltration experiments, recombinant agrobacteria were grown at 29 °C overnight. Cultures were resuspended in infiltration buffer (10 mM Mg_2_SO_4_, 10 mM MES; pH 5.4). Agrobacterium suspension with an optical density (OD_600_) of 0.05 for enzymes and 0.1 for reporters was infiltrated into 4 - to 5 - week-old ∆XTFT plants using a needleless syringe.

### Confocal laser scanning microscopy

To perform subcellular localization studies, leaves of 4 - to 5 - week-old ∆XTFT plants were infiltrated with agrobacterium suspensions carrying the Cma-ST8Sia-GFP fusions at an OD_600_ of 0.05 or 0.1. Confocal images were acquired 2 dpi using an inverted Leica TCS SP8-STED or inverted Zeiss LSM980 with Airyscan2 confocal microscope. The cis-medial Golgi marker MNSI-mRFP was co-infiltrated for colocalization studies^34^. On the Zeiss LSM980, samples were excited using 488 and 561 nm laser lines for GFP and RFP, respectively, and observed in single-track mode using a 63×/1.3 water immersion objective. Signals were detected at 490–534 nm and 588–632 nm for GFP and RFP, respectively. On the Leica SP8, GFP and mRFP were excited simultaneously with the 488 nm and 561 nm laser lines, respectively, and detected at 500–530 nm and 600–630 nm, respectively, using a 63×/1.2 water immersion objective. Post-acquisition image processing was performed in Affinity Photo, LAS X, Zen 3.3 (blue edition) or ImageJ (version 1.54 g).

### Protein extraction and Western blot analysis

Protein extraction and immunoblotting were performed as reported previously with modifications^29^. In short, total proteins (TP) were extracted in extraction buffer (100 mM Tris, 1 mM EDTA, 100 mM NaCl, 40 mM ascorbic acid) containing 0.5% v/v Triton X-100 to extract membrane-bound proteins. The proteins secreted to the intercellular space were collected by isolating intercellular fluid (IF) as described previously^49^. For endoneuraminidase digestion (EndoN; Gift from Rita Gerardy-Schahn), 250 µg of protein extracts (50 µL) were incubated on ice with 0.5 µg of EndoN for 30 min^29,63^. PNGase F (NEB, #P0704S) digestion was carried out according to the manufacturer’s protocol. The samples were heated for 10 min at 65 °C in 4 × Laemmli buffer, and 10 µL samples were resolved on 8% or 12% SDS-PA gel. Gels were used for immunoblotting.

For immunodetection of polySia, nitrocellulose membranes were incubated overnight at 4 °C with anti-polySia mAb735 antibody (1 µg/mL in blocking buffer, i.e., 3% non-fat milk in PBST, pH 7.4) followed by 1 h incubation at RT with 0.2 µg/mL HRP-conjugated goat anti-mouse IgG antibodies in blocking buffer (Promega, #W4021). For immunodetection of VEGFRmFc, 0.2 µg/mL HRP-conjugated anti-human IgG (Invitrogen, #62-8420) for 1 h at RT, Ig5FN1 was detected using 1 µg/mL anti-NCAM antibody 123C3^64^, followed by 0.2 µg/mL goat anti-mouse IgG-HRP. To detect ST8Sia, 0.2 µg/mL anti-strep-HRPO (IBA, #2-1509-001) in blocking buffer was incubated for 1 h. Immunoblots were developed using Clarity Western ECL reagents (Bio-Rad Life Science).

### Glycopeptide analysis by mass spectrometry

The N-glycosylation profile of Ig5FN1 and VEGFRmFc was analyzed by Liquid Chromatography-Electrospray Ionization-Mass Spectrometry (LC-ESI-MS) as previously described^49,65,66^. The 65 KDa VEGFRmFc and 35 KDa Ig5FN1 bands were excised from SDS-PAGE, digested with trypsin, and subjected to LC-ESI-MS using a Thermo Orbitrap Exploris 480. Glycopeptides were identified as peak clusters comprising peptide backbones with N-glycans containing varying numbers of HexNAc, hexose, deoxyhexose, and pentose residues. Manual annotation was performed using FreeStyle 1.8 (Thermo), with spectral deconvolution via the extract function. Relative peak intensities approximated glycoform abundances. Glycan nomenclature is according to^67^.

### EndoN enrichment of polysialylated proteins

To enrich polysialylated protein fractions, IF was isolated from plant leaves expressing Ig5FN1 or VEGFRmFc and mSAP with or without Cma-ST8Sia, as described previously^49^. Approximately 5 mL of IF were incubated on a shaker with 50 µL inactive EndoN coupled CNBr resin for 1.5 h at 4 °C. Subsequently, the liquid was transferred to mini-spin columns (BioRad) and washed with PBS (pH 7.4). The bound polysialylated proteins were eluted in 100 µL elution buffer (100 mM triethylamine, pH 11.5) and neutralized with Tris-HCl buffer (pH 8.0). Ig5FN1-polySia or VEGFRmFc-polySia eluates and intermittent fractions were monitored by Western blot analysis.

### Chain length analysis of polysialic acid

For the chain length analysis, IF containing polysialylated VEGFRmFc was enriched using inactive EndoN coupled to Tosylactivated Dynabeads™ M-280 as described previously^57^. The VEGFRmFc-polySia eluates were dried, and the samples were fluorescently labeled with 1,2-diamino-4,5-methyleneoxybenzene (DMB). Samples were dissolved in 40 µL DMB reagent (2.7 mM DMB (Dojindo, Kumamoto, Japan), 9 mM sodium hydrosulfite, 0.5 M β-mercaptoethanol, 20 mM trifluoroacetic acid) and incubated overnight at 11 °C. The reaction was stopped by adding 10 µL 1 M NaOH, and samples were incubated for 1 h at room temperature to reverse lactonization.

Anion-exchange HPLC analysis was carried out using a Nexera HPLC system (Shimadzu) with a DNAPac PA-100 column (4 × 250 mm; 13 μm; Dionex, Idstein, Germany). The eluents MilliQ water (E1) and 2 N ammonium acetate (E2) were used at a flow rate of 1 mL/min following the gradient t0 min = 0% E2, t5 min = 0% E2, t15 min = 8% E2, t20 min = 11% E2, t35 min = 16% E2, t55 min = 23% E2, t95 min = 31% E2, t130 min = 40% E2, t131 min = 100% E2, t140 min = 100% E2, t141 min = 0% E2, t175 min = 0% E2 to separate polySia chains according to their DP. DMB fluorescence signals were detected using an extinction wavelength of 372 nm and an emission wavelength of 456 nm.

### Microglia inhibition assay

The murine microglial cell line BV2 was cultured and activated as previously described^26^. Briefly, for inflammatory activation, 5 × 10^4^ BV2 cells were seeded in 96-well plates and treated with an activation mix (LLZ) consisting of lipopolysaccharide (LPS, Sigma Aldrich, St. Louis, MO, USA, #L3129, 10 ng/mL), LPS-binding protein (Icell, #C370, 50 ng/mL), and zymosan (InvivoGen, San Diego, CA, USA, #tlrl-zyn, 10 µg/mL) in 200 µL of serum-free medium, and cultured for 24 h in the presence or absence of IF derived polysialylated VEGFRmFc in concentrations 0.1, 1.0, and 10 µg/mL. Nitric oxide (NO) release was determined by the detection of the stable breakdown product nitrite in the cell culture supernatants using the colorimetric Griess assay, and the % inhibition of LLZ-induced NO production is plotted.

### Statistical analysis

GraphPad Prism version 8.02 was used for statistical analysis (GraphPad, San Diego, USA). The one-way ANOVA followed by Tukey’s post-hoc tests was applied as indicated. Normality and equality of variances were assessed using the Shapiro–Wilk and the Brown–Forsythe test, respectively. Values are presented as arithmetic means ± standard error of the mean (SEM); P value < 0.05 was considered statistically significant.

## Supplementary Information

Supplementary Information.

## Data Availability

Data are contained within the article and Supplementary Materials.
