# Supplementary material for: In planta engineering of polysialylated glycoproteins using salmonid polysialyltransferases
Source: Sci Rep. 2026 May 11;16:21531. doi: 10.1038/s41598-026-52259-3 (PMC13350087; doi:10.1038/s41598-026-52259-3)
Supplement: Supplementary file 1 — Supplementary Information. [file 41598_2026_52259_MOESM1_ESM.docx]

**Supplementary Material**

**In Planta Engineering Polysialylated Glycoproteins Using Salmonid Polysialyltransferases**

Lin Sun^1^, Anna Seidel^2^, Hauke Thiesler^3,4^, Jennifer Schoberer^1^, Stanislav Melnik^1^, Alexandra Castilho^1^, Herbert Hildebrandt^3,4^, Anne Harduin-Lepers^5^, Sebastian P. Galuska^2^, Richard Strasser^1^, Rita Gerardy-Schahn^3^, Herta Steinkellner^1^, Somanath Kallolimath^1^*

^1^Institute of Plant Biotechnology and Cell Biology, Department of Biotechnology and Food Sciences, Muthgasse 18, BOKU University, 1190 Vienna, Austria

^2^Research Institute for Farm Animal Biology (FBN), Wilhelm-Stahl-Allee 2, 18196 Dummerstorf, Germany

^3^Hannover Medical School, Institute of Clinical Biochemistry, Carl-Neuberg-Str. 1, Hannover 30625, Germany

^4^Center for Systems Neuroscience Hannover (ZSN), Hannover, Germany

^5^Univ. Lille, CNRS, UMR 8576 - UGSF - Unité de Glycobiologie Structurale et Fonctionnelle, 59000 Lille, France

* Author to whom correspondence should be addressed

Dr. Somanath Kallolimath

Email: Somanath.kallolimath@boku.ac.at

Institute of Plant Biotechnology and Cell Biology, Department of Biotechnology and Food Sciences, Muthgasse 18, BOKU University, 1190 Vienna, Austria

Salmon-ST8SiaII-R1 MQLEFRTVMFGIVTLLVIFLIIADIAEIEEEIANIGGSRTLYLHSLIPKPNRNVAVKANP

Salmon-ST8SiaII-R2 MQLEFRTLMFGIVTVLVIFLIIADIAEVEEEIANIGGSRKLYMHSLIPKPNRNVAVKANP

human-ST8SiaII MQLQFRSWMLAALTLLVVFLIFADISEIEEEIGNSGGRGTIRSAVNSLHSKSNRAEVVIN

***:**: *:. :*:**:***:***:*:****.* ** .: :.: * * .

Salmon-ST8SiaII-R1 TPLISEGEDKSPASPSGLNNTTRLSSDNWTFNRTLSSLIRKNILRFFDPERDISILKGTL

Salmon-ST8SiaII-R2 KPLVSEGEDKSPASPSYSNNTTKLSSDNWTFNRSLSNSIGKNILRFFDPERDISILKGTL

human-ST8SiaII GSSSPAVVDRSNES---IKHNIQPASSKWRHNQTLSLRIRKQILKFLDAEKDISVLKGTL

. . *:* * ::. : :*.:* .*::** * *:**:*:*.*:***:*****

Salmon-ST8SiaII-R1 KPGDVIHYIFDRQSTTNISENLYRLLPTASPMKNQHHRRCAIVGNSGILLNSSCGPEIDS

Salmon-ST8SiaII-R2 KPGDVIHYIFDRQSTTNISENLYRLLPTVSPMKNQHHRHCAIVGNSGILLNSSCGPEIDS

human-ST8SiaII KPGDIIHYIFDRDSTMNVSQNLYELLPRTSPLKNKHFGTCAIVGNSGVLLNSGCGQEIDA

****:*******:** *:*:***.*** .**:**:*. ********:****.** ***:

Salmon-ST8SiaII-R1 HDFVIRCNLAPVEEYAGDVGRRTNLVTMNPSVVQRAFHDLASEQWRERFLQRLRGLSGSV

Salmon-ST8SiaII-R2 YDFVIRCNLAPVEEYAGDVGRRTNLVTMNPSVVQRAFQDLASEEWRERFLQRLRGLSGSV

human-ST8SiaII HSFVIRCNLAPVQEYARDVGLKTDLVTMNPSVIQRAFEDLVNATWREKLLQRLHSLNGSI

:.**********:*** *** :*:********:****.**.. ***::****:.*.**:

Salmon-ST8SiaII-R1 LWIPAFMAKGGEERVEWAIRLILLHTVDVHTAFPSLRLLHAVRGYWLTNNVQIKRPTTGL

Salmon-ST8SiaII-R2 LWIPAFMAKGGEERVEWAIRLILLHTVDVHTAFPSLRLLHAVRGYWLTNNVQIKRPTTGL

human-ST8SiaII LWIPAFMARGGKERVEWVNELILKHHVNVRTAYPSLRLLHAVRGYWLTNKVHIKRPTTGL

********:**:*****. .*** * *:*:**:****************:*:********

Salmon-ST8SiaII-R1 LMYTMATRFCEEIHLYGFWPFPRDSQGIPVKYHYYDTLTYEYTSHASPHTMPLEFRTLSS

Salmon-ST8SiaII-R2 LMYTMATRFCEEIHLYGFWPFPQDSQGKPVKYHYYDTLTYTYTSHASPHTMPLEFRTLSS

human-ST8SiaII LMYTLATRFCKQIYLYGFWPFPLDQNQNPVKYHYYDSLKYGYTSQASPHTMPLEFKALKS

****:*****::*:******** *.: ********:*.* ***:**********::*.*

Salmon-ST8SiaII-R1 LHRQGALRLNTGSCDAGMRS

Salmon-ST8SiaII-R2 LHRQGALRLHTGSCDAGTR-

human-ST8SiaII LHEQGALKLTVGQCDGAT--

**.****:* .*.**..

Salmon-ST8SiaIV MRLSRKRWTICTISILVIFYKTKEITRSEEHQEAQVTGDSELDTSRLMVNSSEKSS-RSG

human-ST8SiaIV MRSIRKRWTICTISLLLIFYKTKEIARTEEHQETQLIGDGELSLSRSLVNSSDKIIRKAG

** **********:*:********:*:*****:*: **.**. ** :****:* ::*

Salmon-ST8SiaIV PSFFQHSVEGWRLNSSLVLMIRKDVLRFLDAERDVSVVKSSFKPGDTIHYVLDRRRTLNI

human-ST8SiaIV SSIFQHNVEGWKINSSLVLEIRKNILRFLDAERDVSVVKSSFKPGDVIHYVLDRRRTLNI

.*:***.****::****** ***::*********************.*************

Salmon-ST8SiaIV SHTLHSLLPDVSPLKNKRFKTCAVVGNSGVLLNSGCGKEIDRHDFVIRCNLAPLAEFAED

human-ST8SiaIV SHDLHSLLPEVSPMKNRRFKTCAVVGNSGILLDSECGKEIDSHNFVIRCNLAPVVEFAAD

** ******:***:**:************:**:* ****** *:*********:.*** *

Salmon-ST8SiaIV VGLRSDFTTMNPSVIQRVYGGLKNATDTERFVQRLRMLNDSVLWIPAFMVKGGERHVESV

human-ST8SiaIV VGTKSDFITMNPSVVQRAFGGFRNESDREKFVHRLSMLNDSVLWIPAFMVKGGEKHVEWV

** :*** ******:**.:**::* :* *:**:** ******************:*** *

Salmon-ST8SiaIV NELIVKRKLRVRTAYPSLRLIHAVRGYWLTNKINIKRPSTGLLMYTLATRFCDEIHLYGF

human-ST8SiaIV NALILKNKLKVRTAYPSLRLIHAVRGYWLTNKVPIKRPSTGLLMYTLATRFCDEIHLYGF

* **:*.**:**********************: **************************

Salmon-ST8SiaIV WPFPRDSNGNVVKYHYYDLLKYRYFSNAGPHRMPLEFKTLKMLHSKGALKLTTSKCESR

human-ST8SiaIV WPFPKDLNGKAVKYHYYDDLKYRYFSNASPHRMPLEFKTLNVLHNRGALKLTTGKCVKQ

****:* **:.******* *********.***********::**.:*******.** .:

**Figure S1: Amino acid sequence alignment of salmonid and human polySTs**. The polybasic region (PBR) and the polysialyltransferase domain (PSTD) are highlighted in grey boxes. ‘*’ identical, ‘:’ strong similarity, ‘.’ Weak similarity, and ‘ ’ blank indicates lack of conservation or mismatch between aligned sequences.


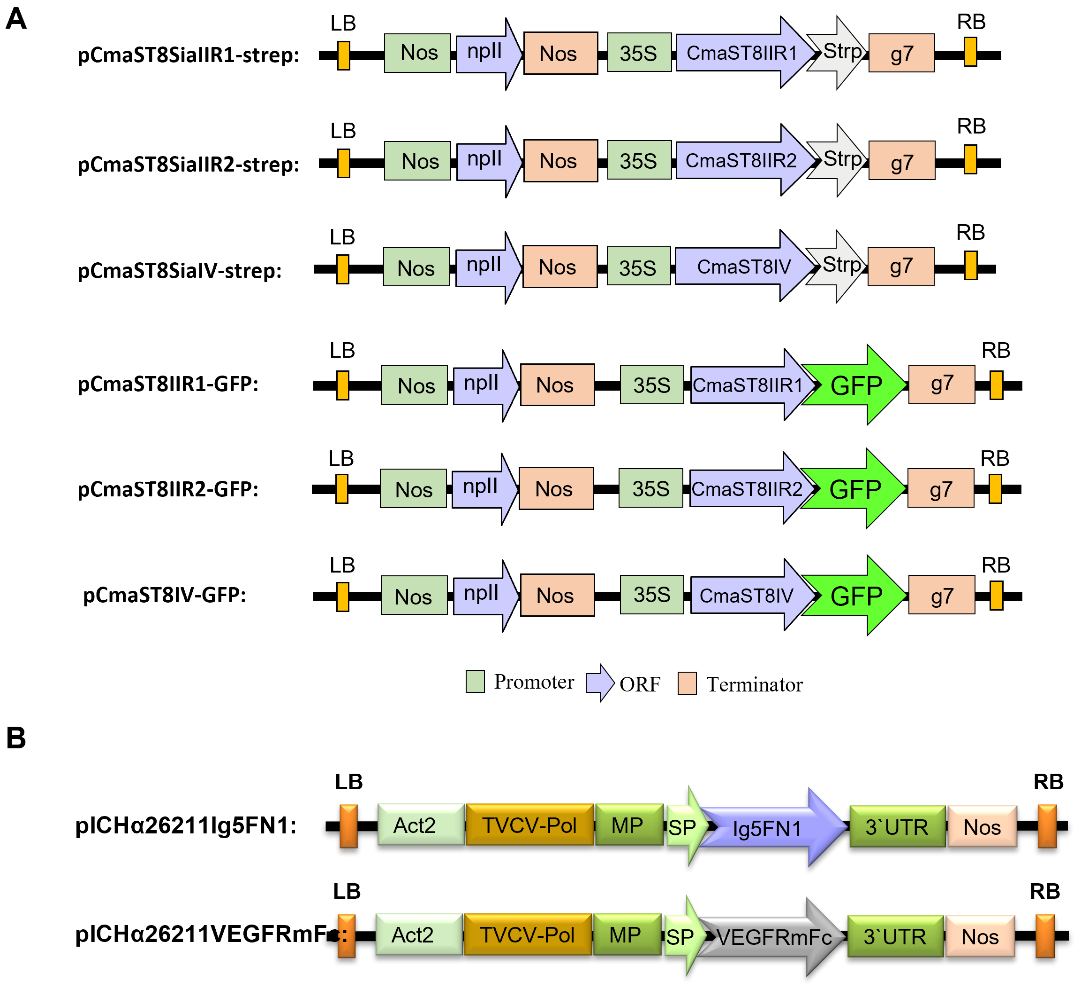


**Figure S2:** **Schematic illustration of the binary vectors**.

**A**) for the expression of CmaST8Sia. LB, the left border; Nos, the nopaline synthase promoter or terminator; npII, the neomycin phosphotransferase II gene that provides resistance to kanamycin; RB, the right border; and CmaST8SiaII-R1, CmaST8SiaII-R2, and CmaST8SiaIV, Strp: Strep II tag; GFP: green fluorescent protein.

**B**) Illustration of magnICON vectors (pICHα26211) for expression of reporter proteins. Ig5FN1 and VEGFRmFc; LB, left border; Act2, Arabidopsis actin 2 promoter; TVCV-Pol, RNA-dependent RNA polymerase from turnip vein-clearing virus; MP, movement protein from TMV; SP, barley α amylase signal peptide, 3′ UTR, 3′-untranslated regions from the tobacco mosaic virus; Nos, nopaline synthase gene terminator; RB, right border.

SDTGRPFVEMYSEIPEIIHMTEGRELVIPCRVTSP**NIT**VTLKKFPLDTLIPDGKRIIWDSRKGFIIS**NAT**YKEIGLLTCEATVNGHLYKTNYLTHRQTNTIIDVVLSPSHGIELSVGEKLVL**NCT**ARTELNVGIDFNWEYPSSKHQHKKLVNRDLKTQSGSEMKKFLSTLTIDGVTRSDQGLYTCAASSGLMTKK**NST**FVRVHEKGGGGSGGGGSLGGPSVFLFPPKPKDTLMISRTPEVTCVVVDVSHEDPEVKFNWYVDGVEVHNAKTKPREEQY**NST**YRVVSVLTVLHQDWLNGKEYKCKVSNKALPAPIEKTISKAKGQPREPQVYTLPPSRDELTKNQVSLRCHVKGFYPSDIAVEWESNGQPENNYKTTKPVLDSDGSFRLYSKLTVDKSRWQQGNVFSCSVMHEALHNHYTQKSLSLSPGK*

VEGFR1-D2-VEGFR2-D3-mFc

**Figure S3: Amino acid (AA) sequence of VEGFRmFc.** VEGFR1‐D2: second domain of human VEGF receptor 1; VEGFR2‐D3: third domain of human VEGF receptor 2; human IgG1 monomeric Fc; stop codon (*), respectively; glycosites are highlighted in red and bold. Underlined AAs refer to Gly-Ser linker.


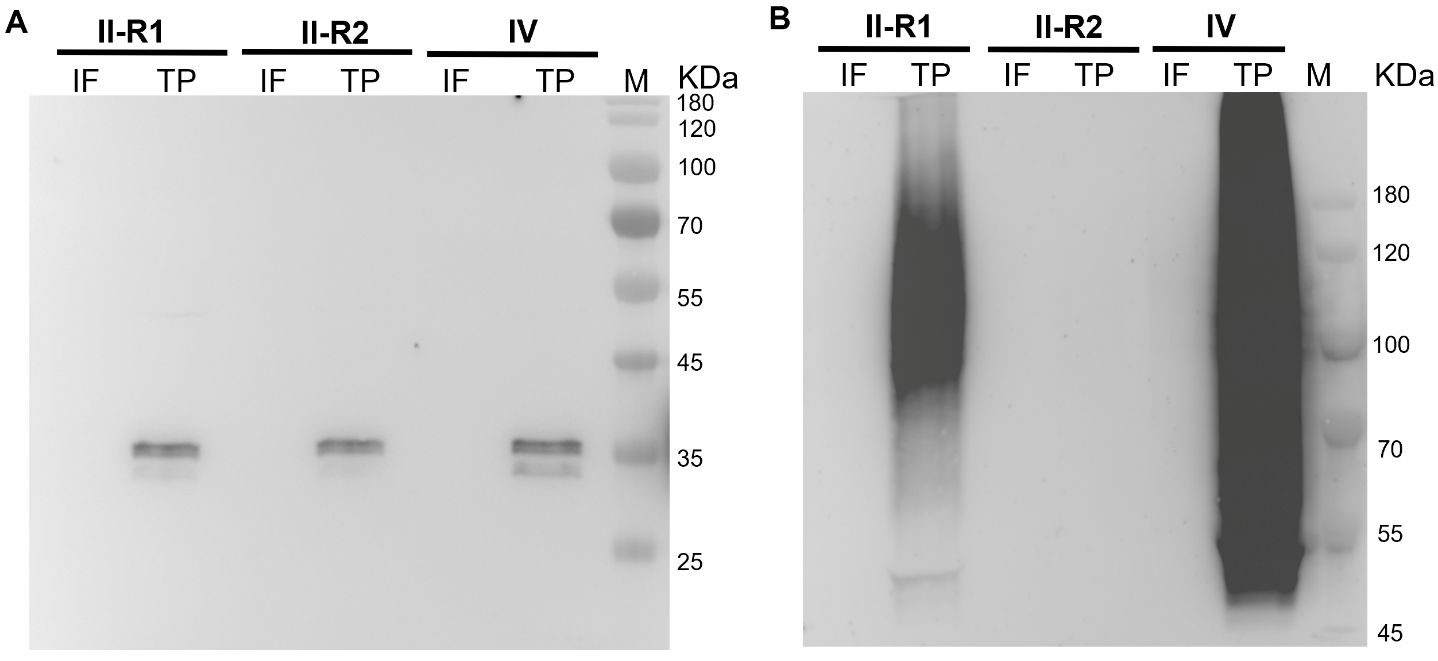


**Figure S4:** **Monitoring of expression and autopolysialylation of CmaST8Sia by Western blot analysis.** 10 µL of intercellular fluid (IF) and total protein (TP) extracted from leaf infiltrated with mSAP and CmaST8Sia (II-R1, II-R2, or IV) were loaded. **A**) anti-strep HRPO antibody, **B**) anti-polySia mAb735 antibody. M: molecular marker.


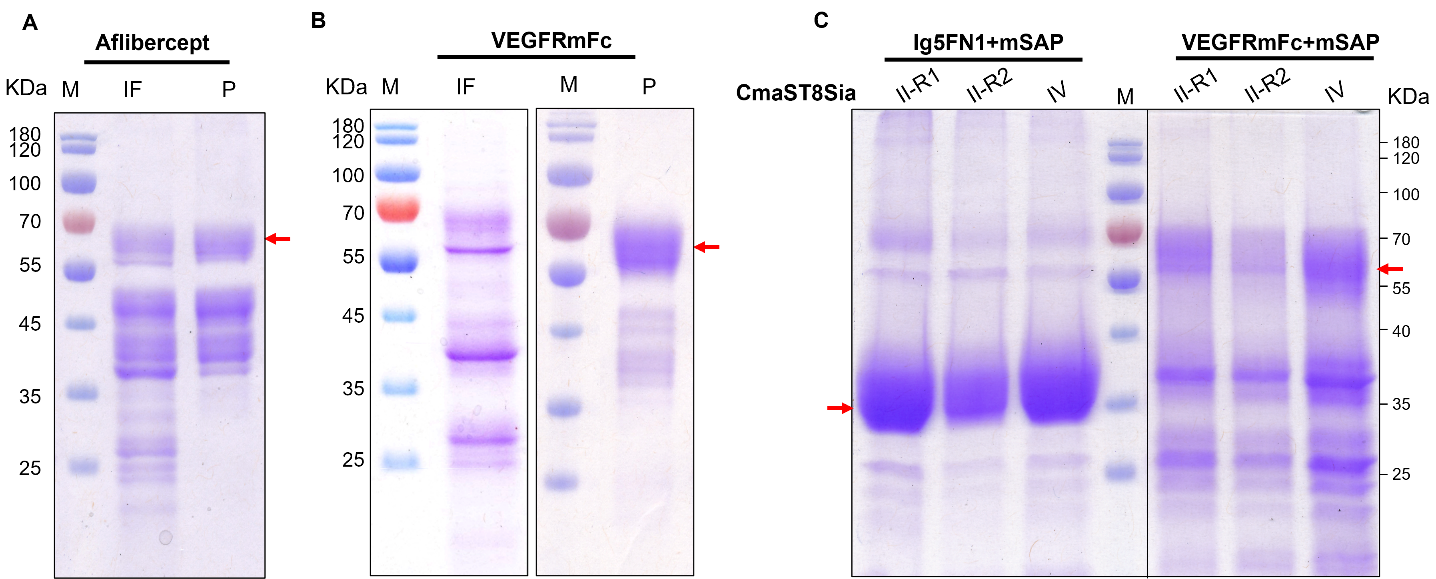


**Figure S5: Monitoring of recombinant Aflibercept, Ig5FN1 and VEGFRmFc using SDS-PAGE.** **A** and **B**) Aflibercept and VEGFRmFc present in intercellular fluid (IF) and protein A purified (P), **C**) IF derived Ig5FN1 and VEGFRmFc co-expressing mSAP and respective CmaST8Sia (II-R1, II-R2 or IV). Arrows indicate the expected size of Aflibercept, VEGFRmFc, and Ig5FN1 (i.e., 70 and 35 kDa, respectively). M: molecular weight marker (KDa); staining: Coomassie Brilliant Blue R-250.


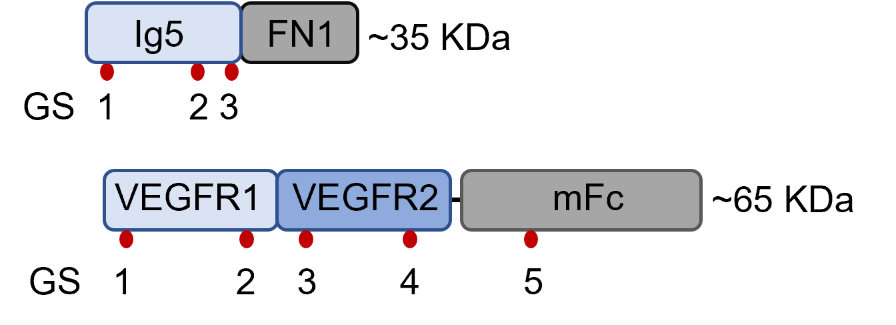


**Figure S6: Schematic illustration of Ig5FN1 and VEGFRmFc, including glycosites (GS).** Theoretical mass is given in KDa. Red dots indicate GS.

**
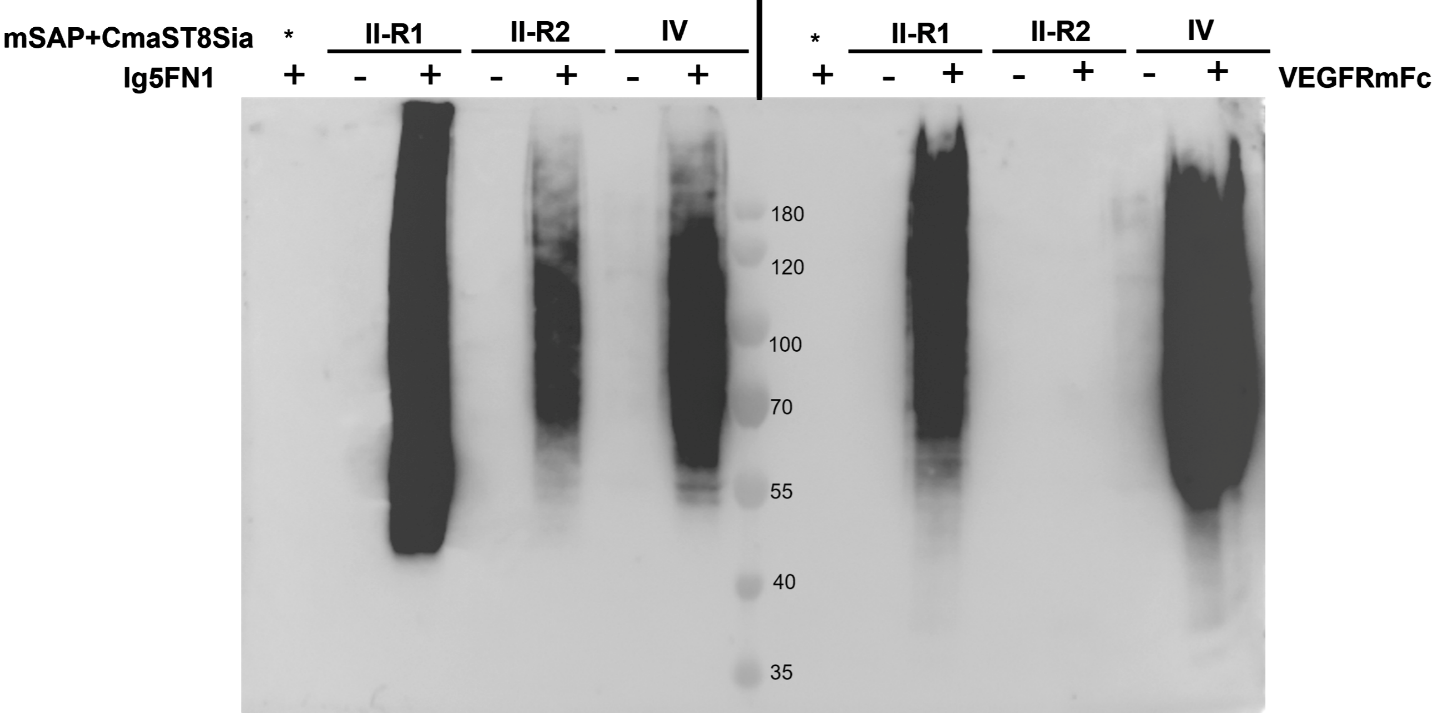
**

**Figure S7:** **Monitoring of polysialylated Ig5FN1 and VEGFR-mFc by Western blot analysis using the** **mAb735 antibody.** 10 µL of intercellular fluid (IF) isolated from leaf infiltrated with mSAP, CmaST8Sia (II-R1, II-R2, or IV), and with (+) and without (-) reporter proteins Ig5FN1 (left) and VEGFRmFc (right) 10µL of IF was loaded. (*) mSAP without Cma-polySTs.


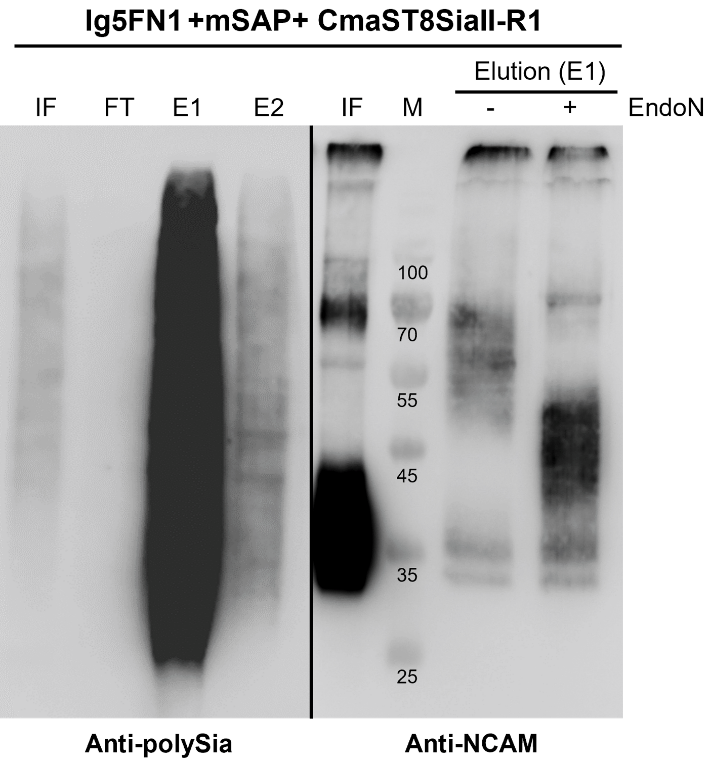


**Figure S8: Monitoring polySia-enriched Ig5FN1 by Western blot analysis using anti-polySia (mAb735) and anti-NCAM (mAb123C3) antibodies, respectively**. 10 µL of samples were loaded on each lane IF: intercellular fluid from leaves expressing Ig5FN1, mSAP, and CmaST8SiaII-R1; FT: Flow through post-incubation with CNBr-coupled inactive Endo N resin. E1 and E2: Elution fractions; M: molecular weight marker; Elution 1 treated without (-) and with (+) Endo N to hydrolyze polysialic acid.

**
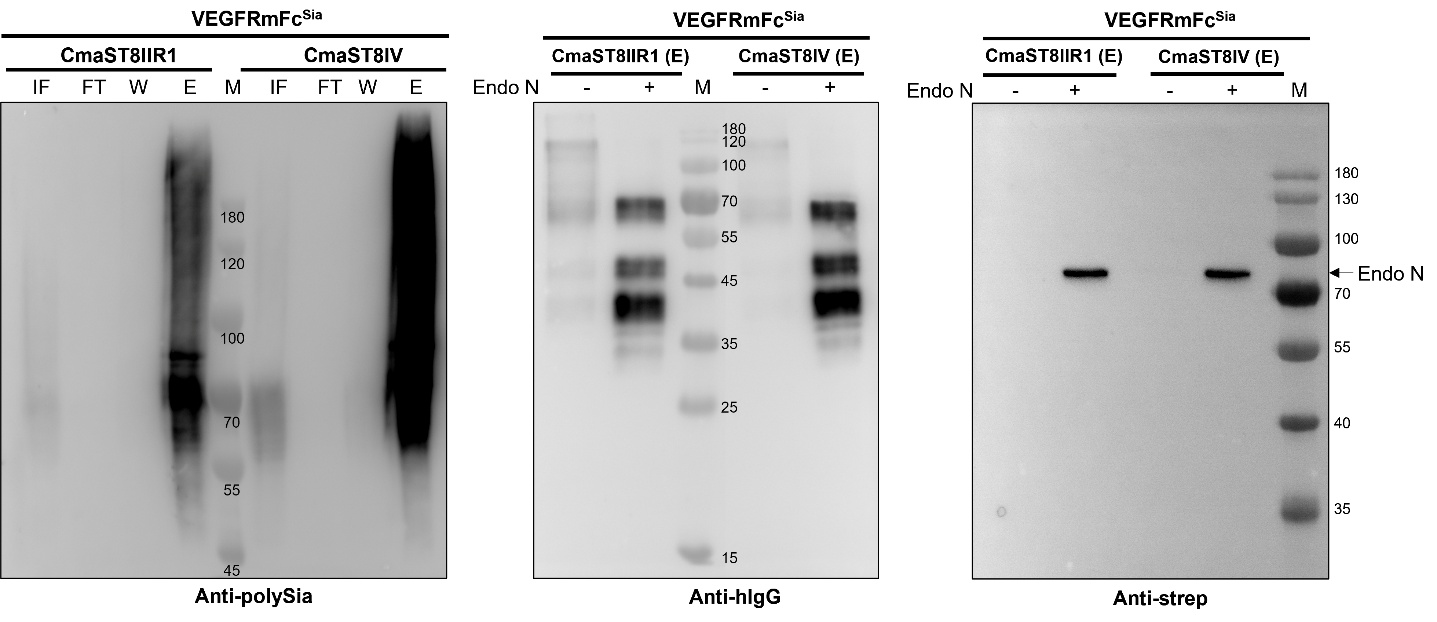
**

**Figure S9: Monitoring polySia-enriched VEGFRmFc by Western blot analysis using anti-polySia mAb735, anti-human IgG, and anti-strep antibodies.** 10 µL of samples were loaded on each lane IF: intercellular fluid from leaves co-expressing VEGFRmFc, mSAP, and CmaST8SiaII-R1 and CmaST8SiaIV; FT: Flow through post incubation with CNBr-coupled inactive Endo N resin. W: wash fraction; E: Elution fraction; M: molecular weight marker; Elution treated without (-) and with (+) Endo N to hydrolyze polySia.


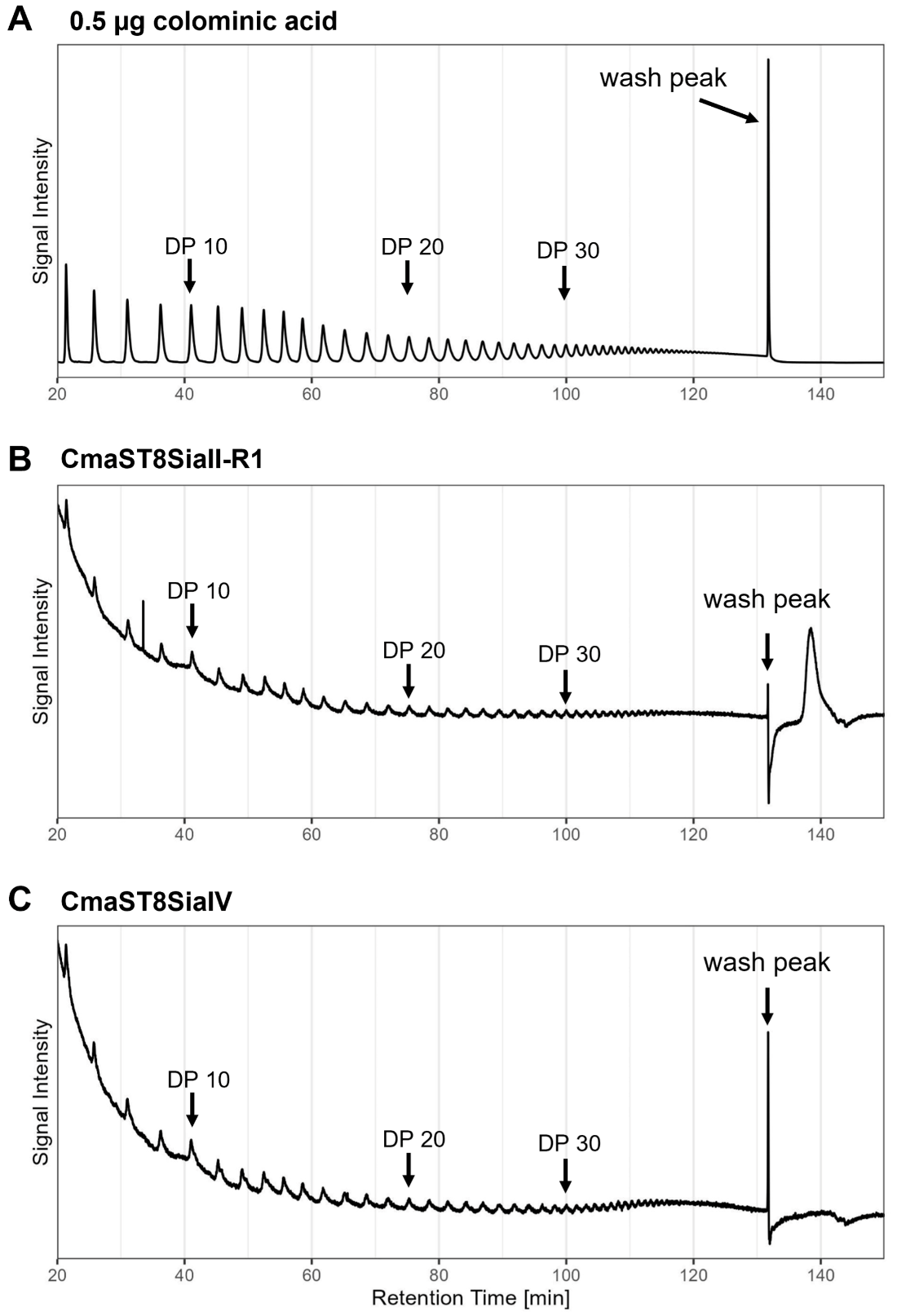


**Figure S10: Chromatograms of chain length analysis of polySia on VEGFRmFc by DMB-anion exchange HPLC.** **A**) 0.5 µg colominic acid was used as a standard to determine the retention times of the DPs. VEGFRmFc polysialylated by **B**) CmaST8II-R1 or **C**) CmaST8IV was isolated using inactive Endo N coupled to magnetic beads and subsequently analyzed by DMB-anion exchange HPLC. For background compensation, the chromatogram of a run with only DMB reagent without any polySia was subtracted from the sample runs. Peaks occurring from column washing are indicated as “wash peak”.


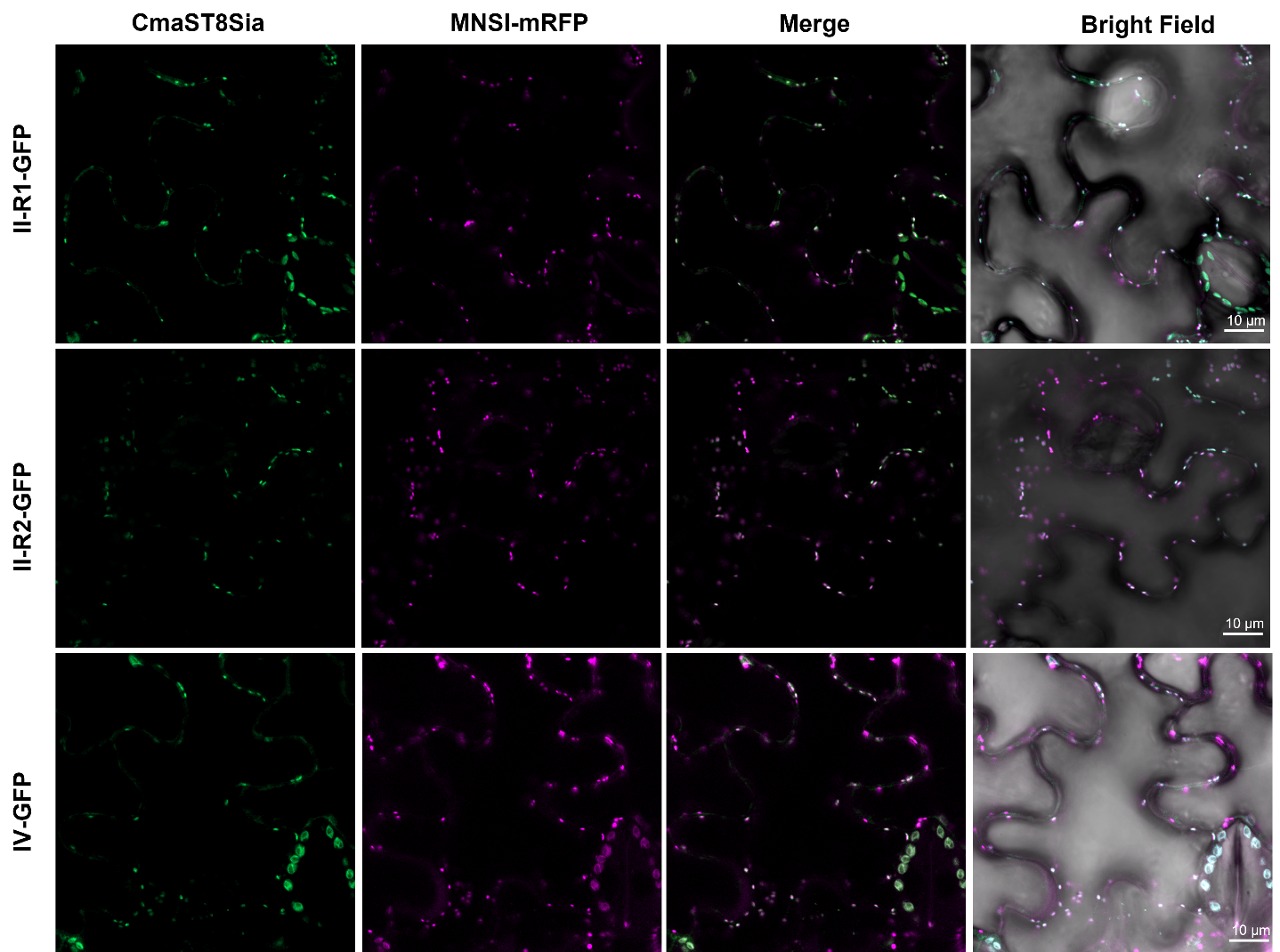


**Figure S11: Subcellular localization of the Cma-ST8Sia-GFP.** Representative confocal images of leaf epidermal cells co-expressing Cma-ST8Sia-GFP fusions and the Golgi marker MNSI-mRFP (mRFP: monomeric red fluorescent protein). The single cell images with a punctate staining pattern of Cma-ST8Sia-GFP (green), which overlaps with that of MNSI-mRFP (magenta), indicate Golgi localization (merge) appears in white, and the corresponding brightfield images outline individual cell boundaries. The Golgi bodies are visualised side-on as they are pushed toward the plasma membrane by the large central vacuole.

**Table S1. Primers used in this study.**

| **Name of the primers** | | **5´------3´** |
| --- | --- | --- |
| CmaST8SiaII-R1_F1 | TATATCTAGAATGCAGTTAGAATTCC | |
| CmaST8SiaII-R1_R1 | TATAAGATCTCATTCCTGCATCAC | |
| CmaST8SiaII-R2_F1 | TATATCTAGAATGCAGTTAGAATTCC | |
| CmaST8SiaII-R2_R1 | TATAAGATCTAGTTCCTGCATCAC | |
| CmaST8SiaIV_F1 | TATATCTAGAATGCGTCTCTCACGG | |
| CmaST8SiaIV_R1 | TATAGGATCCAGATTCGCACTTCG | |

**Table S2. Glycosite (GS) -specific N-glycan distribution of Ig5FN1, excluding unglycosylated fractions.** Nomenclature according to [1].


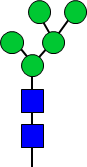

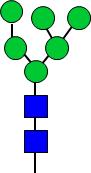

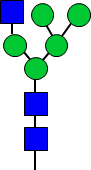

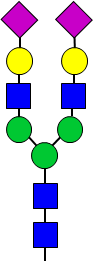

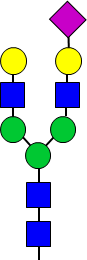

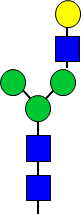

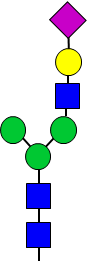

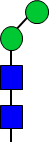

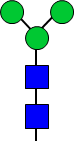

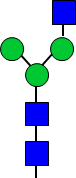

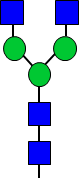

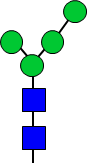

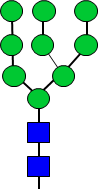

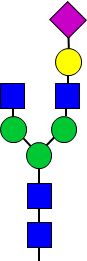

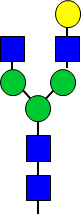

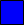


|  | Gn | MU | MM | GnM | GnGn | AM | AGn | NaM | NaGn | NaA | NaNa | Man5Gn | Man4 | Man5 | Man6-9 | Others |
| --- | --- | --- | --- | --- | --- | --- | --- | --- | --- | --- | --- | --- | --- | --- | --- | --- |
| GS1 | 3.3 | 3.5 | 14.0 | 8.5 | 1.1 | 6.0 | 0.0 | **52.3** | **0.0** | **0.0** | **1.1** | 1.8 | 1.6 | 1.6 | 1.9 | 3.2 |
| GS2 | 3.1 | 2.6 | 7.3 | 5.5 | 9.2 | 2.6 | 0.5 | **37.5** | **2.1** | **1.6** | **22.4** | 0.4 | 0.3 | 1.3 | 0.6 | 3.1 |
| GS3 | 0.0 | 2.5 | 7.2 | 5.9 | 11.6 | 2.7 | 0.0 | **44.2** | **1.2** | **0.0** | **23.6** | 0.0 | 0.0 | 0.0 | 0.6 | 0.5 |

**Table S3. Glycosite(GS)-specific N-glycan distribution of VEGFRmFc, excluding unglycosylated fractions.** Nomenclature according [1].


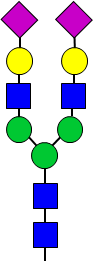

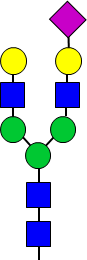

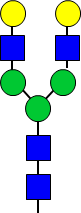

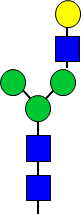

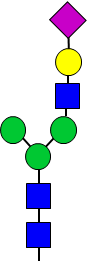

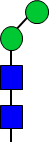

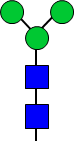

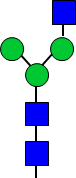

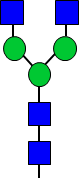

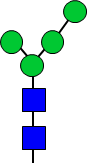

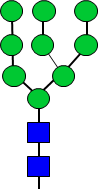

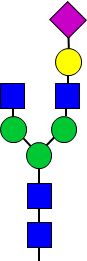

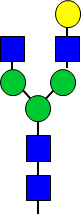

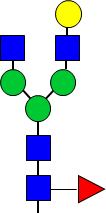

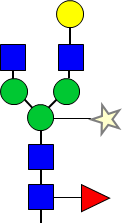

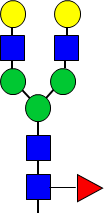

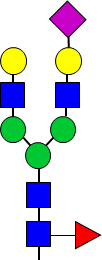

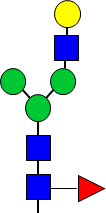


|  |  | MU | MM | GnM | GnGn | AM | AMF | AGn | AGnF | AGnXF | AA | AAF | NaM | NaGn | NaA | NaAF | NaNa | Man4-9 | Others |
| --- | --- | --- | --- | --- | --- | --- | --- | --- | --- | --- | --- | --- | --- | --- | --- | --- | --- | --- | --- |
| GS1 |  | 0.6 | 1.5 | 5.4 | 11.8 | 2.2 | 1.4 | 1.5 | 3.6 | 0.0 | 1.5 | 0.8 | **6.6** | **1.5** | **8.0** | **3.4** | **47.0** | 1.4 | 1.6 |
| GS2 |  | 0.8 | 0.0 | 7.5 | 18.0 | 5.0 | 5.6 | 0.0 | 0.0 | 0.0 | 2.7 | 3.3 | **21.8** | **0.0** | **18.3** | **7.3** | **0.0** | 2.7 | 7.0 |
| GS3 |  | 3.5 | 11.9 | 10.3 | 4.9 | 8.4 | 3.3 | 0.9 | 0.9 | 0.0 | 1.2 | 0.7 | **38.2** | **0.2** | **2.6** | **1.2** | **5.7** | 2.9 | 3.0 |
| GS4 |  | 0.5 | 1.1 | 0.2 | 0.0 | 0.9 | 0.0 | 0.0 | 0.0 | 0.0 | 0.0 | 0.0 | **39.4** | **1.3** | **5.7** | **0.0** | **50.9** | 0.0 | 0.0 |
| GS5 |  | 0.1 | 0.4 | 5.0 | 30.9 | 2.0 | 0.0 | 7.5 | 0.0 | 11.8 | 7.9 | 0.0 | **3.9** | **1.1** | **15.2** | **0.0** | **13.2** | 0.8 | 0.4 |

**Reference:**

1. Altmann, F., J. Helm, M. Pabst, and J. Stadlmann, Introduction of a human- and keyboard-friendly N-glycan nomenclature*.* *Beilstein J Org Chem*. 2024. **20**, p. 607-620.DOI: 10.3762/bjoc.20.53.
